# Low Energy Turnover of Physically Inactive Participants as a Determinant of Insufficient Mineral and Vitamin Intake in NHANES

**DOI:** 10.3390/nu9070754

**Published:** 2017-07-14

**Authors:** Juliane Heydenreich, Katarina Melzer, Céline Flury, Bengt Kayser

**Affiliations:** 1 Swiss Federal Institute of Sport, 2532 Magglingen, Switzerland; juliane.heydenreich@gmail.com (J.H.); katarinamelzer@hotmail.com (K.M.); 2 Institute of Sports Sciences (ISSUL), University of Lausanne, 1015 Lausanne, Switzerland; celine.flury@edu.ge.ch

**Keywords:** total energy expenditure, physical activity level, micronutrients, adults, energy turnover, energy intake, minerals, vitamins

## Abstract

Micronutrient requirements do not scale linearly with physical activity-related energy expenditure (AEE). Inactive persons may have insufficient micronutrient intake because of low energy intake (EI). We extracted data from NHANES 2003–2006 on 4015 adults (53 ± 18 years (mean ± SD), 29 ± 6 kg/m^2^, 48% women) with valid physical activity (accelerometry) and food intake (2 × 24 h-dietary recall) measures. Total energy expenditure (TEE) was estimated by summing the basal metabolic rate (BMR, Harris-Benedict), AEE, and 10% of TEE for the thermic effect of food, to calculate the physical activity levels (PAL = TEE/BMR). Energy intake (EI) was scaled to match TEE assuming energy balance. Adjusted food intake was then analyzed for energy and micronutrient content and compared to estimated average requirements. The NHANES population was physically insufficiently active. There were 2440 inactive (PAL < 1.4), 1469 lightly to moderately active (PAL1.4 < 1.7), 94 sufficiently active (PAL1.7 < 2.0), and 12 very active participants (PAL ≥ 2.0). The inactive vs. active had significantly lower intake for all micronutrients apart from vitamin A, B12, C, K, and copper (*p* < 0.05). The inactive participants had insufficient intake for 6/19 micronutrients, while the active participants had insufficient intake for 5/19 (*p* < 0.05) micronutrients. Multiple linear regression indicated a lower risk for insufficient micronutrient intake for participants with higher PAL and BMI (*p* < 0.001). Symmetrical up-scaling of PAL and EI to recommended physical activity levels reduced the frequency of micronutrient insufficiencies. It follows that prevalence of insufficient micronutrient intake from food in NHANES might be partly determined by low energy turnover from insufficient PAL.

## 1. Introduction

Micronutrients are essential nutrients, required in small quantities for numerous physiological functions [1,2]. They include trace minerals, such as iron, chromium, cobalt, copper, iodine, magnesium, manganese, molybdenum, selenium, and zinc, and also vitamins, which are organic compounds that the organism cannot produce by itself. Micronutrients are essential for health [1,3,4,5], but sub-optimal intake of certain minerals and vitamins is common [5,6]. Micronutrient deficiency can impair cognitive and physical capacities, jeopardize the immune system, and compromise health, in general [1,3,4,7].

Previous studies investigated the adequacy of diet and micronutrient intake recommendations (RDA: recommended daily allowance) [1,2,3,4,5,6,7,8]. An analysis of 70 diets of athletes and non-athletes revealed non-compliance with regard to many compounds [5]. In a European census, several micronutrient-deficient risk groups were identified, including the elderly, pregnant women, vegans, people on a weight reduction diet, and some groups of athletes [3]. In addition, hospitalized and institutionalized people, patients with a chronic inflammatory disorder, participants with chronic administration of certain drugs, and specific clinically-defined patient groups are also considered to be at risk [3]. Although more than two-thirds of the US population is overweight or obese, micronutrient intakes are often found to be below the RDA [9]. Physical activity levels and associated daily energy turnover are recognized to influence micronutrient intake [8]. Csizmadi et al. found that participants with higher physical activity levels have a higher micronutrient intake. They hypothesized that the benefits of higher PALs may extend beyond the usual benefits attributed to physical activity to include having a more favorable impact on adequate nutrient intake [10].

Analyzing the National Health and Nutrition Examination Survey (NHANES) data, Kimmons et al. [6] reported that overweight and obese participants had lower micronutrient intake in comparison with normal weight participants. Since obesity is associated with low physical activity-related energy expenditure [11], this finding raises the question of whether increased energy expenditure, in conjunction with increased energy intake, would improve compliance with micronutrient intake recommendations. Physical activity bouts can, depending on fitness level, increase energy expenditure up to >20 times the basal metabolic rate (BMR) [12]. Regular physical activity is, therefore, accompanied by increased total energy expenditure and, in order to achieve energy balance, with increased dietary intake [1,13,14,15]. According to Melzer et al. [16], over longer periods, energy intake normally follows moderate to vigorous physical activity energy requirements for activities cumulatively lasting two or more hours per day.

Contrary to the energy requirements, the micronutrient requirements of inactive and physically active persons are quite similar [14]. For athletes, who typically have high energy expenditure and intake, even though there may be an increased need for some compounds, there is generally no need for supplementation [14,17]. This is essentially due to greater overall dietary intake, to cover the increased physical activity-related energy expenditure, coupled with an often enhanced food quality observed in more active participants [18,19,20].

In this study, we explored the extent to which physical activity levels of a sample of the US adult population are associated with compliance with dietary intake recommendations for minerals and vitamins. We also explored by how much of an increase in physical activity levels, up to levels recommended for health, combined with a corresponding linear up-scaling of dietary intake without altering dietary composition, would improve compliance with recommended micronutrient intake.

## 2. Materials and Methods

We extracted data from NHANES, a continuing population-based survey conducted by the Centers for Disease Control and Prevention, that uses a complex, stratified, multi-stage probability sample design in order to create a representative sample of the civilian, non-institutionalized U.S. population [21,22]. The National Center for Health Statistics ethics review board approved the protocols, and written informed consent was obtained from all NHANES participants. Anonymous data are freely available for analysis on the NHANES repository [23]. For our study, we needed quantification of energy and micronutrient intakes and an objective measurement of physical activity. Two data collection periods satisfied these conditions and were used for the analysis: NHANES 2003–2004 and NHANES 2005–2006.

### 2.1. Analytical Sample

We combined NHANES 2003–2004 and NHANES 2005–2006 data files to obtain a first sample with 20,470 participants. Of this sample, 10,081 participants were asked to wear an accelerometer, and 7139 provided valid measures of physical activity by use of accelerometry. We then excluded participants younger than 21 years (*n* = 2778, to exclude any late growth), pregnant women (*n* = 180) [24,25], participants without anthropometrical measurements (*n* = 25), and participants without dietary recall (*n* = 129). According to Westerterp [26], in free-living humans, the physical activity level (PAL) ranges between 1.1 and 2.5. Technical artifacts from accelerometry can lead to erroneously extreme PALs. To minimize the errors, we excluded 12 participants with a PAL lower than 1.1 and greater than 2.5 from the sample. Thus, the analytical sample contained 4015 participants (53 ± 18 years (mean ± SD), 81 ± 20 kg, 29 ± 6 kg/m^2^), of which 1945 (48%) were women. The datasets analyzed during the current study are available from the corresponding author on reasonable request.

### 2.2. Dietary Intake

The nutritional assessment component of NHANES included two 24 h dietary recalls. The first was conducted in person by trained dieticians in a mobile examination center using a standard set of measuring guides to help the respondent report the volume and dimensions of the food items consumed. Upon completion of the in-person interview, participants were given measuring cups, spoons, a ruler, and a food model booklet to use for reporting food amounts for a second 24 h recall through telephone interview. The telephone interviews were collected 3–10 days following the in-person interview, on a different day of the week. Dietary macro and micronutrient compositions and quantities were calculated with standard food tables (USDA Food and Nutrient Database for Dietary Studies, 2.0). The processed data (in SAS format) were downloaded from the NHANES website [23]. The average energy and nutrient intake over the two days for each participant was used for the present analysis. The NHANES sodium intake included all sources of salt, including that from table salt.

### 2.3. Energy Expenditure

Activity energy expenditure was measured with an accelerometer (Actigraph AM-7167, Pensacola, FL, USA) in a one-minute epoch setting. The device was carried on the right hip attached to an elastic band. Participants were asked to carry the device for seven days, to keep the device dry (i.e., remove it before swimming or bathing), and to remove the device at bedtime. Data collection occurred between the first and during and/or after the second 24 h dietary intake recalls. We downloaded the raw accelerometer count data (in SAS format) from the NHANES website and used the SAS programs published by the National Cancer Institute to reduce the data [27]. Energy expenditure from physical activity was then estimated with the Williams transformation [28]:

Kcals = CPM × 0.0000191 × BM(1)

where Kcals = total calories for a single epoch, CPM = counts per minute, and BM = body mass (kg). The mean wearing time of the accelerometers was 14.3 ± 1.8 h per day (range: 10–23 h per day).

BMR was calculated using the Harris-Benedict equation [29]. We estimated total energy expenditure (TEE) by summing BMR and daily physical activity energy expenditure estimated from the accelerometer data, adding a further 10% to account for the thermic effect of food [30]. We then calculated physical activity level (PAL = TEE/BMR). The data were analyzed separating the participants into groups according to their PAL: inactive (PAL < 1.4), moderately active (PAL 1.4 ≤ 1.7). and active participants (PAL > 1.7). The chosen classification was adapted from the established classification provided by the World Health Organization (WHO) [31].

### 2.4. Micronutrients

We considered 19 micronutrients: 10 vitamins (A, B1, B2, B3, B6, B9, B12, C, E, and K) and nine minerals (calcium, phosphorus, magnesium, iron, zinc, copper, sodium, potassium, and selenium).

Daily intakes were compared to the dietary reference intakes provided by the Food and Nutrition Board of the Institute of Medicine in the USA [32,33,34,35,36]. The individual intake was compared to the estimated average requirement (EAR) for most of the micronutrients. For those micronutrients where no EAR is established (vitamin K, potassium, and sodium) the individual intake was compared to the adequate intake (AI). Individual micronutrient intake was also compared to the tolerable upper intake levels (UL). Fortification of certain foods with vitamins B12 and E was included in the total vitamin intake. Any supplements were not taken into account in order to only describe micronutrient intake from food sources.

### 2.5. Data Analysis

In a first step, we analyzed original dietary intake data and compared it to US dietary intake recommendations. Since we found that the reported energy intakes did not, on average, cover the estimated energy expenditures, we corrected for the estimated energy deficits, assuming energy balance and under-reporting by NHANES participants, as suggested by Archer et al. [37]. Energy balance was expressed as energy intake (kcal/day) minus TEE (kcal/day) and as the quotient between energy intake and BMR. We linearly increased (or decreased) nutrient intake data so that energy intake matched TEE, without changing diet composition. The corrected values were then compared to the dietary intake recommendations again. Finally, we increased all individual PALs that were <2.0, up to a PAL of 2.0. In parallel, we linearly increased dietary intake, without changing diet composition, to quantify the resulting changes in micronutrient intake and compliance with recommendations for daily micronutrient intake. For those participants, where the initial PAL was ≥2.0 (*n* = 12) the dietary intake was decreased in order that energy intake matched energy expenditure with a PAL of 2.0.

Lastly, assuming a fixed energy cost of 0.93 kcal/kg per km for level brisk walking, we transformed the necessary increase in individual energy expenditure to bring PAL up to 2.0 into an increased daily walking distance, since walking is the principal means for increasing physical activity in inactive people [38].

### 2.6. Statistical Analysis

Accelerometer data was transformed using SAS version 9.3 (SAS Institute, Cary, NC, USA) using the code developed by the National Cancer Institute [27]. All further data analysis was performed using SPSS Statistics version 23.0 (IBM Corporation, Armonk, NY, USA). Normality was checked using the Kolmogorov-Smirnov-test. Not all data were normally distributed and their analysis was performed with non-parametric tests. Mann-Whitney U-tests were used to perform sex comparisons, and Kruskal-Wallis tests were used to assess differences between PAL groups. Spearman-Rho correlations were performed to assess the relationships between various variables. We used multiple linear regression analysis with the forced entry method in order to quantify the relationship between chosen independent (number of insufficient vitamin, mineral, and micronutrient intake) and dependent variables (age, sex, PAL, and BMI). The alpha level cut-off was set at 0.05.

## 3. Results

### 3.1. Characteristics of the Participants

The participants’ characteristics are described per sex (Table 1) and per PAL (Table 2). The weight, height, and BMI of males were significantly higher compared to females (*p* < 0.01).

The adult NHANES population is on average insufficiently physically active. There were 2440 (very) inactive (PAL < 1.4; 52.7% females), 1469 lightly to moderately active (PAL 1.4 - < 1.7; 43.3% females), 94 sufficiently active participants (PAL 1.7 - < 2.0; 20.2% females), and 12 very active (PAL ≥ 2.0; 25.0% females). Inactive participants were significantly older than moderately active and active participants (*p* < 0.05). There was a significant negative correlation between age and PAL (*r* = −0.44, *p* < 0.0001).

### 3.2. Energy Balance

Sufficiently and very active participants (PAL ≥ 1.7) showed a greater absolute and relative negative energy balance compared to inactive and lightly to moderately active participants (*p* < 0.05; Table 3). There was a significant negative correlation between PAL and absolute and relative energy balance (*r* = −0.15 and *r* = −0.12, respectively; all *p* < 0.0001). The ratio of energy intake and BMR was higher in sufficiently and very active participants compared to inactive and lightly to moderately active participants (*p* < 0.05).

Obese participants (BMI ≥ 30) showed a higher absolute and relative negative energy balance and a lower ratio of energy intake and lower BMR compared to all other BMI subgroups (*p* < 0.0001; Table 4). There was a significant negative correlation between BMI and absolute and relative energy balance (*r* = −0.37 and *r* = −0.35, respectively; all *p* < 0.0001). In addition, a significant negative correlation between BMI and the ratio of energy intake and BMR was observed (*r* = −0.34, *p* < 0.0001).

Those participants whose baseline micronutrient intakes were compliant with the recommendations, defined as having micronutrient intakes above the EAR or AI (*n* = 130), having a significantly higher ratio of energy intake and BMR than those participants with at least one micronutrient intake not meeting the requirements (1.9 ± 0.6 vs. 1.3 ± 0.5, *p* < 0.0001).

A linear up-scaling in energy intake to cover a theoretical increase in PAL to 2.0 for all participants with a PAL < 2.0 would require an increase of an additional 13.4 ± 3.1 km (range: 0.1–23.9 km) of daily brisk walking, on average.

### 3.3. Micronutrient Intake

Female participants had a significantly lower intake of all micronutrients, apart from vitamin K, compared to male participants (*p* < 0.01, Table 5). Male participants had also a lower total number of insufficient micronutrient intakes compared to female participants (5.2 ± 3.2 micronutrients (3.5 ± 2.1 vitamins and 1.6 ± 1.5 minerals) vs. 5.9 ± 3.9 micronutrients (3.9 ± 2.4 vitamins and 2.0 ± 1.8 minerals); *p* < 0.001).

Inactive participants had a lower intake of all micronutrients compared to lightly to moderately active participants, with significant differences for all micronutrients apart from vitamin A and vitamin K (all *p* < 0.05; Table 6). Furthermore, inactive participants had a lower intake of all micronutrients compared to sufficiently and very active participants, with significant differences for all micronutrients, apart from vitamins A, B12, C, K, and copper (all *p* < 0.05). Sufficiently and very active participants showed a lower total number of insufficient micronutrient intakes compared to inactive participants (4.9 ± 3.6 micronutrients (3.4 ± 2.2 vitamins and 1.5 ± 1.6 minerals) vs. 5.8 ± 3.6 micronutrients (3.9 ± 2.3 vitamins and 2.0 ± 1.6 minerals); *p* < 0.05).

When nutrient intake was adapted so that energy intake matched estimated total energy expenditure, inactive participants had a lower intake of all micronutrients compared to moderately active participants, with significant differences for all micronutrients apart from vitamin K (all *p* < 0.01), and lower intake compared to active participants, with significant differences for all micronutrients apart from vitamins K and B12 (all *p* < 0.01). Inactive participants had less insufficient micronutrient intakes compared to moderately active and active participants (4.9 ± 2.6 micronutrients (3.4 ± 1.8 vitamins and 1.5 ± 1.2 minerals) vs. 3.7 ± 2.2 micronutrients (2.8 ± 1.6 vitamins and 0.9 ± 1.0 minerals) and 3.1 ± 1.9 micronutrients (2.5 ± 1.4 vitamins and 0.6 ± 0.9 minerals); *p* < 0.05). Male participants had less insufficient micronutrient intakes compared to female participants (4.3 ± 2.4 micronutrients (3.1 ± 1.6 vitamins and 1.2 ± 1.1 minerals) vs. 4.6 ± 2.7 micronutrients (3.2 ± 1.9 vitamins and 1.3 ± 1.2 minerals); *p* < 0.001).

**Figure 1:**
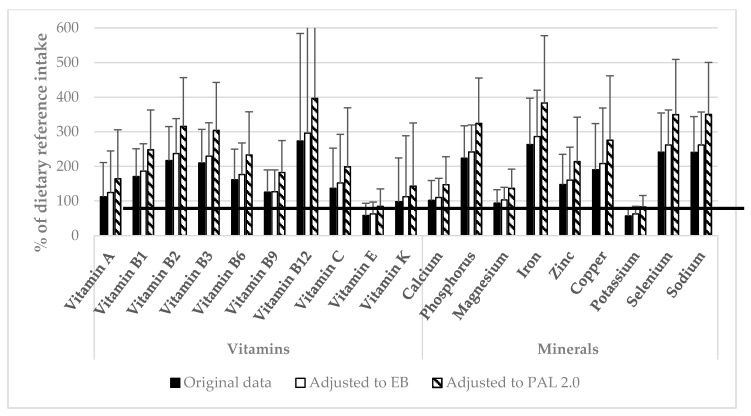
Vitamin and mineral intake in percentage of dietary reference intake (adequate intake for vitamin K, potassium, and sodium; estimated average requirement for the remaining micronutrients) for original data (black bars), data adjusted for energy balance (EB, white bars), and data adjusted for physical activity level (PAL) of 2.0 (shaded bars). The solid line represents 100% of the dietary reference intake. Data are shown as mean ± SD.

In Figure 1, the vitamin and mineral intake in percentage of the dietary reference intake is displayed. The mean intakes of vitamin E (58.3%), vitamin K (98.2%), magnesium (93.9%), and potassium (57.2%) were below the recommendations. When data were adjusted to reach energy balance, the mean intake of vitamin E (62.7%) and potassium (62.6%) were still below recommendations. When data were adjusted to a PAL of 2.0, intakes were still below recommendations for vitamin E (84.5%) and potassium (83.0%).

For some micronutrients intake greatly exceeded recommendations. For the EB-adjusted dataset, mean intake of sodium was 3606 ± 1375 mg/day (262% of AI), while it reached 5205 ± 1884 mg/day (350%) after the adjustment to a PAL of 2.0. More than 80% of participants (85.6%, *n* = 3436) had an intake above the UL in the EB-adjusted dataset, whereas when the data was adjusted to a PAL of 2.0 the intake was above UL for 93% of participants (*n* = 3715). For vitamin B3, 17% of the participants (*n* = 697) had an intake above UL (EB-adjusted dataset), and when data were adjusted to a PAL of 2.0 intake was above UL for 42% of the participants (*n* = 1704).

**Figure 2:**
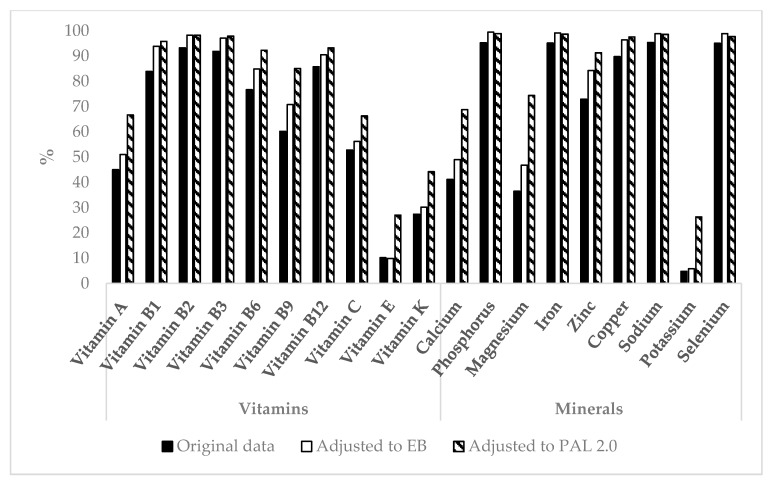
Percentage of participants with sufficient vitamin and mineral intake. Black bars indicate original data, white bars show adjusted data for energy balance (EB), and shaded bars indicate data adjusted for physical activity level (PAL) of 2.0.

For vitamin E, vitamin K, magnesium, and potassium only 10.2%, 27.4%, 36.5%, and 4.8% of participants had sufficient intake, respectively (Figure 2). When original data was adjusted to a PAL of 2.0, intake of vitamin E, vitamin K, magnesium, and potassium was sufficient in 27.0%, 44.2%, 74.4%, and 26.3% of participants, respectively. There was no sex difference in the average sum of insufficient micronutrient intakes (*p* = 0.76).

When energy and nutrient intake was adjusted so that energy intake matched total energy expenditure, the sum of insufficient vitamin intakes was significantly associated with age (β = –0.04, *p* = 0.03), BMI (β = −0.22, *p* < 0.001), and PAL (β = −0.21, *p* < 0.001), but not with sex (β = 0.01, *p* = 0.47). The adjusted R^2^ for the model was 0.09. The sum of insufficient vitamin intakes was lowest in older participants with a higher BMI and a higher PAL. In addition, the sum of insufficient mineral intakes was significantly associated with sex (β = 0.04, *p* < 0.05), age (β = 0.14, *p* < 0.001), BMI (β = −0.27, *p* < 0.001), and PAL (β = −0.26, *p* < 0.001), with an adjusted R^2^ for the model of 0.20; and was lowest in younger, male participants with a higher BMI and a higher PAL.

## 4. Discussion

The main findings of this study are (1) NHANES nutritional intake data underestimate actual intake and need to be adjusted before interpretation; (2) NHANES participants with higher physical activity levels were more in line with recommendations for mineral and vitamin intake compared to insufficiently active participants; and (3) modeling an increase in physical activity to higher levels, together with a linear up-scaling of food intake with the same dietary composition, to compensate for the increased energy expenditure, increased compliance with recommendations for micronutrient intake. These findings underline how levels of physical activity, through the effect on energy intake, impact on the intake of non-energy constituents for a given diet composition. The lack of physical activity comes with an increased risk of mineral and vitamin deficiencies, as hypothesized. Our modelling would further suggest that increasing physical activity levels might be protective.

There was a negative correlation between PAL and the number of insufficient micronutrient intakes (*r* = −0.14, *p* < 0.0001). The participants whose baseline micronutrient intakes were compliant with the recommendations, defined as having an intake above the EAR or AI (*n* = 130), had a significantly higher ratio of energy intake and BMR compared to those with at least one micronutrient intake not meeting the recommendations. Non-compliance with the recommendations might be related to a higher magnitude of underreporting (low ratio of energy intake and BMR). On the other hand, those participants with complete compliance of micronutrient intake had PALs ranging from 1.13 to 2.03. This suggests that not only PAL and, hence, energy intake, play a role, but also diet composition. In that respect, the increased energy turnover due to increased physical activity could have an additional favorable impact on nutrient adequacy if it is accompanied by changes in dietary composition and/or supplementation with certain minerals and vitamins. Similar conclusions were drawn by other large-scale studies as well [10].

In an analysis of NHANES III data (1988–1994), Kimmons et al. [6] reported that participants who were overweight or obese, particularly premenopausal women, were more likely to report low levels of micronutrient intake (particularly vitamins E, C, and D, beta-carotene, selenium, and folate) than were normal-weight participants in the same sex/age category. These results are in line with findings from other studies that assessed the relationship between obesity and micronutrient intake [6,40,41,42,43,44,45,46,47,48,49].

NHANES dietary intake values might not be accurate, because of the data collection method used a 2 × 24 h dietary recall method. This, and other techniques to quantify eating habits, lack accuracy, with reported underestimations of intake up to 20%, in particular in obese individuals [50]. Briefel et al. [51] analyzed the NHANES III data (1988–1994) and reported that dietary intake was probably underestimated in up to 18% of men and 28% of women. Archer et al. [37] analyzed NHANES data (from 1971 through 2010) by using physiologically-credible energy intake values, and estimated an average under-reporting of 281 and 365 kcal/day for men and women, respectively, with greater under-reporting for participants with a greater BMI. In our NHANES data sample, we were able to actually estimate under-reporting of intake, since the objective measurement of daily physical activity with accelerometers in NHANES 2003–2006 allowed us to estimate the physical activity-related energy expenditure and to calculate the energy balance. Our results suggest NHANES 2003–2006 dietary intake data are underreported by an average of 176 and 109 kcal/day for women and men, respectively, with greater under-reporting for those with a higher BMI (*r* = 0.34, *p* < 0.0001) and higher physical activity levels (*r* = 0.12, *p* < 0.0001).

An increase in physical activity levels is not necessarily immediately compensated by an energetic equivalent increase in food intake. The type and duration/intensity of physical activity, as well as the body composition of individuals, when they engage in more physical activity, be it in the form of physical activity integrated into daily life (walking, cycling, stair climbing), exercise (jogging, working out), or sports, affect food intake regulation and its changes over time. We previously reported that overweight or obese untrained participants who engage in a long-term physical activity program do not necessarily increase energy intake during the first months [16,52]. This absence of an immediate compensatory increase in food intake in the obese might be due to their excess energy stores. Fully compensatory responses in intake to altered levels of exercise energy expenditure might not begin before a certain amount of the excess adipose tissue is depleted. Conversely, more active and lean individuals would have to increase their energy intake in response to a further increase in physical activity to prevent weight loss.

We chose to model the effect of a linear up-scaling of dietary intake to cover the energy requirements of a PAL= 2.0. A PAL of 1.7 identifies participants who can be considered to be minimally sufficiently physically active while a PAL of 1.9 may be necessary to prevent weight gain over time [53]. However, a PAL around 2.0 is more representative of typical behavior observed in modern hunter-gatherers and may reflect habitual *Homo sapiens* activity for most of its history [54]. It likely is a level sufficiently high to lead to eventual compensatory responses in food intake, but is obviously challenging to implement in modern, everyday life. It would imply a change of an entire lifestyle that is in contrast to the one supported by “modern” life in motorized and food-abundant surroundings. We calculated that, on average, the NHANES population would need to walk briskly for an additional 13 ± 3 km per day, something difficult to envisage in the USA at present. Other means to increase PAL, such as more non-exercise physical activity into daily occupational, transportation and household routines, were proven to be a useful strategy for increasing energy expenditure in otherwise inactive participants [55,56,57], although it is acknowledged that a meaningful increase in energy turnover is plausible only at high PALs in lean participants performing physical activity on a regular basis [16,52].

Increasing PAL to 2.0 increased mean intakes of all vitamins and minerals by 56–66%. However, the higher energy turnover did not fully correct imbalances of all minerals and vitamins. For example, the baseline sodium intake of our sample was, on average, more than double that of the EAR [36]. A linear up-scaling of sodium intake further increased the already worrisome sodium intake levels, which could jeopardize health and lead to an potential increase in cardiovascular risk [58]. More than 90% of the participants would have a sodium intake above the UL after data adjustment. On the other hand, intake of vitamin E and potassium still remained below recommendations when linear up-scaling was performed.

Our study has limitations. The participants were not equally distributed when they were divided into the three PAL subgroups (PAL < 1.4, PAL 1.4 - < 1.7, and PAL ≥ 1.7), but were composed of 60.8% (*n* = 2440), 36.6% (*n* = 1469), and 2.6% (*n* = 106) of the participants, respectively. Furthermore, the determination of PAL was based on the results from accelerometer data, which are prone to recording and/or analysis errors. Accelerometers have low sensitivity in low-intensity activities and are unable to register static exercise nor the activities that do not involve a transfer of the center of mass at a rate relative to the energy expended (e.g., weight lifting, uphill walking, walking and carrying a load) [59]. In addition, there is currently no consensus related to the selection of cut-off points to define activity intensities despite a number of proposed cut-offs for some devices. Furthermore, the TEE was estimated using formulas and not objectively measured with methods, such as doubly-labeled water.

Published studies using NHANES 2003–2004 data have reported that 5% of adults performed 30 minutes or more of physical activity on a daily basis [60]. Our analyses show that only 2.6% of participants were compliant with a PAL ≥ 1.7, which corresponds to a daily physical activity of moderate intensity of approximately 45–60 min, in order to prevent unhealthy weight gain [53].

Evaluation of nutritional intake has some methodological weaknesses, such as misreporting or under-reporting, that limit the interpretation of dietary record data. The NHANES dietary intake was analyzed using the 2 × 24 h dietary recall technique, which is subject to bias. In order to be able to exclude data that might not be authentic, Archer et al. [37] suggested using a ratio of energy intake and BMR that is less than 1.35 to identify the values that seem implausible. Our analyses showed energy intake to BMR ratios of 1.26 ± 0.44, 1.37 ± 0.50, and 1.52 ± 0.58 for inactive, moderately active, and active participants, respectively. However, 59.8% of all participants had a ratio of energy intake and BMR of less than 1.35. This, again, raises the question of whether memory-based dietary assessment methods should be used for the assessment of energy and nutrient intake [61,62,63].

We further did not take into account that under-reporting of dietary intake is not necessarily consistent across the various constituents of a diet. For instance, it was reported that fat may be more under-reported than other food constituents [64], which would be of relevance for fat-soluble vitamins. In addition, the nutritional analysis in NHANES derives mineral and vitamin intake from food tables according to the declared intake and not from a direct analysis of daily food intake.

Finally, our modeling strategy applied the assumption that an increase in physical activity-related energy expenditure would be automatically compensated by a reciprocal increase in food intake without changes to dietary composition. This theoretical model likely oversimplifies the true associations (physical activity vs. linear up-scaling in diet quantities and composition), which may be non-linear, and also dependent on the obesity status of participants.

The strength of our study lies in the fact that we used a large dataset in which physical activity was measured objectively. The chosen study model may serve as a baseline for future studies, which can deal with the aforementioned limitations and investigate them in more detail using a longitudinal study design.

## 5. Conclusions

Even after correcting for inadequate dietary intake reporting there is a high prevalence of insufficient micronutrient intake in the adult NHANES population. Prevalence is higher in participants with lower PALs. Insufficient mineral and vitamin intake thus seems partly determined by low energy turnover from insufficient PALs. An increase in the population’s PALs might lead to increased energy intake to cover the increased expenditure and, at the same time, increased intake of the non-energy compounds in food, like minerals and vitamins, reducing the prevalence of insufficient mineral and vitamin intake.

## Figures and Tables

**Table 1:** Characteristics of included participants differentiated by sex.

Participants	*n*	Age (Years)	Weight (kg)	Height (cm)	BMI (kg/m^2^)
Males	2070	52.5 ± 17.9	86.5 ± 18.7 ^a^	175 ± 8 ^a^	28.3 ± 5.3
Females	1945	52.8 ± 17.5	74.8 ± 19.0	161 ± 7	28.9 ± 6.9
Total	4015	52.7 ± 17.7	80.8 ± 19.7	168 ± 10	28.6 ± 6.1

Data are shown as mean ± SD. BMI = body mass index; ^a^ significantly different to females (*p* < 0.001).

**Table 2:** Characteristics of included participants differentiated by physical activity level (PAL).

PAL	*n*	Age (Years)	Weight (kg)	Height (cm)	BMI (kg/m^2^)
<1.4	2440	57.2 ± 18.3 ^a^	79.4 ± 20.0 ^b^	167 ± 10 ^a^	28.4 ± 6.2 ^b^
1.4- <1.7	1469	45.9 ± 14.2	83.1 ± 19.0	169 ± 10	28.4 ± 5.1
≥1.7	106	42.5 ± 13.9	82.1 ± 19.1	170 ± 9	28.9 ± 6.1

Data are shown as mean ± SD. BMI = body mass index; ^a^ significantly different to PAL groups 1.4 ≤ 1.7 and ≥ 1.7 (*p* < 0.05); ^b^ significantly different to PAL group 1.4 ≤ 1.7 (*p* < 0.01).

**Table 3:** Energy balance in kcal/day and percentage of total energy expenditure (TEE) differentiated by physical activity level (PAL).

PAL	*n*	Energy Intake (kcal/Day)	Energy Balance		EI/BMR
			kcal/Day	% of TEE	
<1.4	2440	1942 ± 731 ^a^	−78 ± 690 ^a^	−2.5 ± 33.8 ^a^	1.26 ± 0.44 ^a^
1.4- <1.7	1469	2286 ± 904	−216 ± 847	−8.0 ± 33.2	1.37 ± 0.50
≥1.7	106	2589 ± 1003 ^b^	−574 ± 1041 ^b^	−16.9 ± 31.6 ^b^	1.52 ± 0.58 ^b^

Data are shown as mean ± SD. Energy balance was calculated as energy intake (EI; kcal/day)–TEE (kcal/day). Basal metabolic rate (BMR) was calculated by use of Harris-Benedict equation [27]. ^a^ significantly different to PAL groups 1.4 ≤ 1.7 and ≥ 1.7 (*p* < 0.0001); ^b^ significantly different to PAL group 1.4 ≤ 1.7 (*p* < 0.05).

**Table 4:** Energy balance in kcal/day and percentage of total energy expenditure (TEE) differentiated by Body Mass Index (BMI).

BMI (kg/m^2^)	*n*	Energy Balance		EI/BMR
		kcal/Day	% of TEE	
<18.5	55	529 ± 816 ^a^	31.9 ± 46.9 ^a^	1.74 ± 0.63 ^a^
18.5- <25	1144	175 ± 712 ^b^	9.4 ± 35.6 ^b^	1.49 ± 0.49 ^b^
25- <30	1462	−136 ± 693 ^c^	−6.0 ± 30.1 ^c^	1.30 ± 0.43
≥ 30	1354	−443 ± 762	−17.2 ± 29.2	1.14 ± 0.41

Data are shown as mean ± SD. Energy balance was calculated as energy intake (EI; kcal/day)–TEE (kcal/day). Basal metabolic rate (BMR) was calculated by use of the Harris-Benedict equation [29]. BMI was classified according to standard WHO classification [39]. ^a^ Significantly different to all other BMI groups (*p* < 0.05); ^b^ significantly different to BMI groups 25 - < 30 and ≥ 30 (*p* < 0.01); ^c^ significantly different to BMI group ≥ 30 (*p* < 0.01).

**Table 5:** Micronutrient intake (original data without dietary supplement intake) of included participants differentiated by sex.

			Males (*n* = 2070)		Females (*n* = 1945)	
			Intake	DRI *	Intake	DRI *
**Vitamins**	Vitamin A	[µg/day]	677 ± 660 ^a^	625	584 ± 454	500
		[µg/MJ]	71.6 ± 78.4 ^a^		83.2 ± 66.8	
	Vitamin B1	[mg/day]	1.9 ± 0.9 ^a^	1.0	1.9 ± 0.8	0.9
		[mg/MJ]	0.19 ± 0.07 ^a^		0.20 ± 0.07	
	Vitamin B2	[mg/day]	2.5 ± 1.2 ^a^	1.1	1.9 ± 0.8	0.9
		[mg/MJ]	0.26 ± 0.10 ^a^		0.26 ± 0.10	
	Vitamin B3	[mg/day]	28.2 ± 12.9 ^a^	12	20.1 ± 8.4	11
		[mg/MJ]	2.9 ± 1.0		2.8 ± 1.0	
	Vitamin B6	[mg/day]	2.2 ± 1.1 ^a^	1.1	1.7 ± 0.8	1.1
		[mg/MJ]	0.23 ± 0.10		0.24 ± 0.11	
	Vitamin B9	[µg/day]	446 ± 222 ^a^	320	354 ± 173	320
		[µg/MJ]	45.8 ± 18.5 ^a^		49.8 ± 22.0	
	Vitamin B12	[µg/day]	6.4 ± 7.5 ^a^	2.0	4.5 ± 4.2	2.0
		[µg/MJ]	0.66 ± 0.84		0.63 ± 0.59	
	Vitamin C	[mg/day]	96.5 ± 83.5 ^a^	75	86.9 ± 72.6	60
		[mg/MJ]	10.1 ± 8.7 ^a^		12.5 ± 11.1	
	Vitamin E	[mg/day]	7.6 ± 4.4 ^a^	12	6.3 ± 3.9	12
		[mg/MJ]	0.77 ± 0.35 ^a^		0.87 ± 0.49	
	Vitamin K	[µg/day]	105 ± 141	120	99 ± 121	90
		[µg/MJ]	11.2 ± 16.8 ^a^		14.5 ± 21.7	
**Minerals**	Calcium	[mg/day]	948 ± 509 ^a^	800	789 ± 398	800
		[mg/MJ]	96.2 ± 40.1 ^a^		110 ± 48	
	Phosphorus	[mg/day]	1473 ± 586 ^a^	580	1112 ± 419	580
		[mg/MJ]	149 ± 34 ^a^		154 ± 37	
	Magnesium	[mg/day]	321 ± 131 ^a^	350	254 ± 105	265
		[mg/MJ]	33.0 ± 9.9 ^a^		35.7 ± 11.7	
	Iron	[mg/day]	18.0 ± 8.5 ^a^	6.0	13.7 ± 6.3	8.1
		[mg/MJ]	1.9 ± 0.7 ^a^		1.9 ± 0.8	
	Zinc	[mg/day]	13.9 ± 9.0 ^a^	9.4	10.0 ± 5.3	6.8
		[mg/MJ]	1.4 ± 0.9		1.4 ±0.7	
	Copper	[mg/day]	1.5 ± 1.1 ^a^	0.7	1.2± 0.7	0.7
		[mg/MJ]	0.15 ± 0.13 ^a^		0.16 ± 0.09	
	Potassium	[mg/day]	2990 ± 1152 ^a^	4700	2365 ± 878	4700
		[mg/MJ]	309 ± 92 ^a^		334 ± 105	
	Selenium	[µg/day]	124 ± 55 ^a^	45	92 ± 40	45
		[µg/MJ]	12.6 ± 3.8		12.8 ±4.1	
	Sodium	[mg/day]	3781 ± 1644 ^a^	1500	2825 ± 1101	1500
		[mg/MJ]	382 ± 113 ^a^		392 ± 104	

DRI = dietary reference intake. Data are shown as mean ± SD. * For all micronutrients, apart from vitamin K, potassium, and sodium the estimated average requirement (EAR) for the age group 31–50 years is displayed. For vitamin K, potassium, and sodium the average intake is shown. ^a^ significantly different from females (*p* < 0.01).

**Table 6:** Micronutrient intake (original data without dietary supplement intake) of included participants differentiated by PAL.

Micronutrient Intake		PAL		
		<1.4 (*n* = 2440)	1.4 ≤ 1.7 (*n* = 1469)	≥1.7 (*n* = 106)
**Vitamins**	Vitamin A [µg/day]	625 ± 516	644 ± 658	636 ± 503
	Vitamin B1 [mg/day]	1.6 ± 0.7 ^a^	1.8 ± 0.9	1.9 ± 0.8 ^b^
	Vitamin B2 [mg/day]	2.1 ± 1.0 ^a^	2.3 ± 1.1	2.4 ± 1.1
	Vitamin B3 [mg/day]	22.9 ± 11.1 ^a^	26.5 ± 12.2	27.6 ± 11.4
	Vitamin B6 [mg/day]	1.9 ± 1.0 ^a^	2.1 ± 1.1	2.2 ± 1.1
	Vitamin B9 [µg/day]	385 ± 197 ^a^	424 ± 215	459 ± 219
	Vitamin B12 [µg/day]	5.3 ± 5.9 ^b^	5.8 ± 6.8	5.7 ± 3.9
	Vitamin C [mg/day]	88 ± 74 ^b^	97 ± 84	107 ± 97
	Vitamin E [mg/day]	6.6 ± 4.0 ^a^	7.5 ± 4.5	7.8 ± 4.7
	Vitamin K [µg/day]	100 ± 124	104 ± 135	121 ± 220
**Minerals**	Calcium [mg/day]	824 ± 431 ^a^	942 ± 502	968 ± 555
	Phosphorus [mg/day]	1219 ± 496 ^a^	1412 ± 578	1543 ± 703
	Magnesium [mg/day]	273 ± 115 ^a^	310 ± 129	344 ± 163
	Iron [mg/day]	15.3 ± 7.3 ^a^	16.9 ± 8.4	17.3 ± 8.5
	Zinc [mg/day]	11.5 ± 8.3 ^a^	12.8 ± 6.6	13.4 ± 6.5
	Copper [mg/day]	1.3 ± 0.8 ^a^	1.5 ± 1.2	1.5 ± 0.7
	Potassium [mg/day]	2580 ± 994 ^a^	2831 ± 1146	3167 ± 1433
	Selenium [µg/day]	103 ± 47 ^a^	117 ± 54	133 ± 63 ^b^
	Sodium [mg/day]	3141 ± 1377 ^a^	3578 ± 1598	3766 ± 1670

Data are shown as mean ± SD. PAL = physical activity level. ^a^ significantly different from PAL groups 1.4 ≤ 1.7 and ≥ 1.7 (*p* < 0.05); ^b^ significantly different from PAL group 1.4 ≤ 1.7 (*p* < 0.05).
